# Phytoavailability of bound residue of Carbendazim to Chinese cabbage *(Brassica campestris ssp*.*chinensis*) coexisted with Superabsorbent polymers

**DOI:** 10.1038/s41598-020-57488-8

**Published:** 2020-01-16

**Authors:** Yatian Yang, Haiyan Wang, Wei Li, Yan Chen, Wei Guo, Xin Gu, Qingfu Ye

**Affiliations:** Institute of Nuclear Agricultural Sciences, Key Laboratory of Nuclear Agricultural Sciences of Ministry of Agriculture and Zhejiang Province, Zhejiang University, Hangzhou, 310029 China

**Keywords:** Environmental impact, Nutrition

## Abstract

Understanding the bioavailability and phytotoxicity of Carbendazim (MBC) bound residues (BR) in soils incubated with different Superabsorbent polymer (SAP) amendment on succeeding crops is essential to assess their environmental fate and risks. In our research, we studied the morphological characteristics and ^14^C-accumulation of Chinese cabbage and released BR in three typical cultivated soils. The plant dry weight was in order of superabsorbent-hydrogels formulations (HMBC) > MBC > MBC and SAP (MBC-SAP) at 35 d in basic soil 3 (S3), with 675.40 ± 29.07 mg/plant.d.w, 575.93 ± 25.35 mg/plant.d.w and 427.86 ± 18.79 mg/plant.d.w. The whole plant accumulated 2-fold more BR when grew in neutral soil 2 (S2) treated with SAP than MBC at 7 d. The root accumulated a greater proportion of ^14^C-MBC residue than shoot, with order of MBC-SAP > MBC > HMBC at 21d. The results indicate MBC-BR could be released and accumulated in plant. HMBC promoted the Chinese cabbage growth with lowest ^14^C accumulation, while MBC-SAP inhibited plant growth with the highest ^14^C uptake. The released BR rate was 61.43 ± 3.75% of initial BR in MBC-SAP, with 2-fold higher than MBC and HMBC. It is assumed HMBC could be a potential environmentally friendly measure for rational use of pesticides in future.

## Introduction

Pesticides are ubiquitous chemicals in the environment, and are usually used to control crop disease and maintain the products. When pesticides are applied to the field and undergo degradation, their metabolites and they could bind to organic or mineral constituents of soil, and form the non-extractable residue (bound residue) in soil^1^. Bound residue (BR) is generally regarded as the soil detoxification process, which involves chemicals could permanently bind to soil matrix, and are no longer bioavailable and bio-accessible to organisms. However, some studies reported that BR could be released via microbial activity and physic-chemical mechanism, and accumulated in living tissues and food webs, posing high threat to human health^2^. Han *et al*. found ^14^C-labeled residue of ZJ0273 was released from the BR-amended soil upon planting, and rice seedling took up the ^14^C from soil contaminated with compound residues and was inhibited to grow^3^. Gao *et al*. detected a clear uptake, accumulation and translocation of phenanthrene and pyrene by ryegrass, and significant phytoavailabilty of bound-PAH residue^4^. Liu *et al*. also demonstrated that the earthworms accumulated 31.5% of the total radioactivity ^14^C-BR-RM^5^ after exposure to the BRs of ^14^C-CYC. Thus, the food safety issue of agricultural products originated from contaminated soils should be given a public concern. The potential release, bioaccumulation and phytotoxicity of the BR to succeeding non-target organism are always important topics for understanding the environment and human food safety impact of pesticides.

Soils are the vital resources that provide life-supporting services of food production, water cleaning and habits for human and wildlife. Intensified agricultural production has deteriorated the soil quality and led to the increasing amount of anthropogenic contaminants to the environment. The contamination of agricultural soils has a plethora of negative impacts on food production and agroecosysterm service. Soil amendments or water adsorbents are applied in the agriculture to realize the coupled effects of water, fertilizer and pesticides. Superabsorbent polymer (SAP) is common soil conditioner to hold the soil moisture and improve soil property in agriculture^6–8^. Moreover, SAP also has been employed in combination with pesticides as a new formulation to control their release rates and to promote the efficient use of both pesticides and water^9–11^. Therefore, SAP is usually coexisting with pesticides in agriculture in different forms of amendments or new pesticides formulations. However, relatively less work has been conducted to study the soil environmental fate of pesticides when amended with SAP. Yang *et al*. found that when SAP coexisted with pesticides in soil, the BR of MBC was increased when soil spiked with MBC and SAP (MBC-SAP), and decreased in term of superabsorbent hydrogels (SHs) formulations (HMBC)^12,13^. Nowadays, the potential for the release and subsequent availability and phytotoxic effects of BR when pesticides coexist with SAP or encapsulate as new formulations remain poorly understood, especially for the succeeding crops and food contamination in agriculture production. In fact, the bioaccumulation of pesticides amended with SAP or SHs-formulations in plants, especially crops, can cause potential risks in the food chain and human health. Therefore, it is important to clarify the bio-effect of pesticides-bound residue to crops and to provide safe agricultural products.

Chinese cabbage is a popular green leafy crop, and is widely vegetated for its rich nutritional and favorable taste. The shoot and leaf can be eaten at the seedling stage, and the seed can be contacted into soil at the ripening stage. Chinese cabbage seedlings are also common succeeding crops in fields. MBC, a broad spectrum benzimidazole fungicide, is usually used against fungi which affect crops, fruits and vegetables. However, MBC can be accumulated in plant and transferred to different parts, interfere mitosis of bacterial cell and inhibit its growth^14,15^. For example, Alicja Lewandowska *et al*. found that MBC residue can be taken up by plants from extractable residue in soils^16^. Thus, the primary objective of this study is to evaluate the plant availability and phytotoxicity of MBC-BR after the amendment with SAP to Chinese cabbage seedlings. We planted the Chinese cabbage seed into the MBC-BR amended soil with HMBC, MBC-SAP and MBC treatment to reveal the effect of BR on the growth of Chinese cabbage by measuring the plant height, plant weight, tracing the ^14^C content distribution and accumulation patterns in the different part of Chinese cabbage for safety assessment. Furthermore, we detected the BR release rate, extractable residue and bound residue in soil after the Chinese cabbage cultivation to demonstrate the soil safety.

## Results

### The effects of BR on cabbage growth

The growth parameters of cabbage, including plant height, dry weight of shoot, root, flower, and total plant were determined to evaluate the soil BR effects on plant growth (Table 1). Cabbage gradually grew with the incubation time in all treatments. Significant difference was observed in the growth of cabbage between three tested soils, indicating cabbage growth may be closely related with soil property and microbes. Cabbage didn’t have a favorable growth in S_1_ and grew till 21 d. The dry weight of total plant was all below 5.00 mg for each plant, and the height of whole plant was around 1.50 cm in all treatments, which was significantly lower than those in S_2_ and S_3_ (*p* < 0.05). Meanwhile, there was no significant difference between HMBC, MBC-SAP, MBC treatment and blank control in S_1_ (*p* > 0.05).

**Table 1:** Effects of BR of ^14^C-Carbendazim on the growth of cabbage in soil.

Soil	Treatment	Time (d)	Plant height (cm)	Dry weight of shoot (mg/plant)	Dry weight of root (mg/plant)	Dry weight of flower (mg/plant)	Total weight (mg/plant)
S_1_	Control	7	1.25 ± 0.08a	2.07 ± 0.32abcde	0.73 ± 0.15b	0.00 ± 0.00a	2.27 ± 0.06ade
		21	1.12 ± 0.03b	1.40 ± 0.40acde	2.80 ± 1.84ad	0.00 ± 0.00a	3.33 ± 1.50abdef
	MBC	7	1.32 ± 0.06a	2.13 ± 0.40abcde	0.67 ± 0.15b	0.00 ± 0.00a	2.80 ± 0.53acdef
		21	1.22 ± 0.06a	2.43 ± 0.51bce	1.63 ± 0.25a	0.00 ± 0.00a	4.07 ± 0.76bcdf
	MBC + SAP	7	1.49 ± 0.06c	1.70 ± 0.52cde	0.97 ± 0.12d	0.00 ± 0.00a	2.67 ± 0.64def
		21	1.36 ± 0.11ca	1.30 ± 0.53de	2.10 ± 0.79a	0.00 ± 0.00a	3.40 ± 0.89def
	HMBC	7	1.50 ± 0.19acd	1.77 ± 0.06de	0.70 ± 0.10b	0.00 ± 0.00a	2.47 ± 0.15e
		21	1.25 ± 0.28abcd	2.13 ± 0.78e	1.63 ± 0.25a	0.00 ± 0.00a	3.77 ± 0.99 f
S_2_	Control	7	4.15 ± 0.17a	11.55 ± 0.23a	1.90 ± 0.10a	0.00 ± 0.00a	13.45 ± 0.19a
		21	10.82 ± 1.01b	79.50 ± 2.36b	13.20 ± 1.05b	0.00 ± 0.00a	92.70 ± 2.36b
		35	24.15 ± 0.17c	149.47 ± 14.20c	82.03 ± 4.31c	20.70 ± 0.79b	252.20 ± 18.22c
	MBC	7	5.08 ± 0.21d	12.97 ± 2.45a	1.90 ± 0.44a	0.00 ± 0.00a	14.87 ± 2.32a
		21	8.82 ± 0.92e	86.37 ± 2.83d	13.50 ± 1.15b	0.00 ± 0.00a	97.73 ± 5.62b
		35	45.06 ± 0.07 f	365.23 ± 11.31e	139.93 ± 15.77d	38.83 ± 4.83c	544.00 ± 15.70d
	MBC + SAP	7	6.42 ± 0.47 g	13.17 ± 7.15a	2.03 ± 0.15a	0.00 ± 0.00a	15.20 ± 7.14a
		21	11.09 ± 0.09b	79.97 ± 1.53b	10.80 ± 2.19b	0.00 ± 0.00a	92.77 ± 3.89b
		35	37.32 ± 0.59 h	398.27 ± 17.59 f	268.70 ± 9.46e	37.47 ± 3.93c	704.43 ± 16.17e
	HMBC	7	7.27 ± 0.50 g	13.17 ± 3.87a	2.33 ± 0.78a	0.00 ± 0.00a	15.50 ± 4.60a
		21	12.17 ± 0.33k	184.03 ± 16.12 g	11.20 ± 1.95b	0.00 ± 0.00a	195.23 ± 16.49 f
		35	31.81 ± 1.19 l	571.97 ± 21.40 h	305.70 ± 14.63 f	52.43 ± 8.70d	930.10 ± 22.89 g
S_3_	Control	7	3.28 ± 0.20a	8.73 ± 0.45a	1.33 ± 0.21a	0.00 ± 0.00a	10.07 ± 0.46a
		21	11.15 ± 0.17bj	75.23 ± 5.17b	6.10 ± 4.25aceg	0.00 ± 0.00a	81.34 ± 1.01b
		35	14.15 ± 0.17c	292.30 ± 13.49c	131.12 ± 12.28b	0.00 ± 0.00a	446.93 ± 35.55c
	MBC	7	4.18 ± 0.16d	11.83 ± 2.89a	1.50 ± 0.70ac	0.00 ± 0.00a	13.33 ± 3.56a
		21	8.82 ± 0.37e	77.53 ± 6.28b	10.73 ± 0.67c	0.00 ± 0.00a	88.27 ± 0.76d
		35	15.06 ± 0.07 f	270.67 ± 14.71c	305.27 ± 14.95d	0.00 ± 0.00a	575.93 ± 25.35e
	MBC + SAP	7	5.92 ± 0.45 g	11.10 ± 3.94ad	1.50 ± 0.53a	0.00 ± 0.00a	12.60 ± 3.55a
		21	10.42 ± 0.49hj	72.60 ± 5.24b	7.87 ± 0.32e	0.00 ± 0.00a	80.47 ± 4.34b
		35	18.32 ± 0.59i	301.43 ± 18.04c	110.73 ± 7.07 f	15.70 ± 0.82b	427.86 ± 18.79c
	HMBC	7	4.61 ± 0.42d	6.53 ± 1.07d	1.23 ± 0.25a	0.00 ± 0.00a	7.77 ± 0.95a
		21	11.17 ± 0.93j	114.40 ± 15.94e	4.73 ± 0.42 g	0.00 ± 0.00a	119.13 ± 8.31 f
		35	25.14 ± 0.35k	443.43 ± 23.50 f	231.97 ± 6.21 h	0.00 ± 0.00a	675.40 ± 29.07 g

On the contrary, cabbage underwent an appreciate growth in S_2_ and S_3_ and blossomed at 35 d. There was a time-dependent biomass increase trend during the whole incubation. Compared with blank control, the growth of cabbage was promoted in all treatments spiked with MBC-BR in S_2_. Moreover, the dry weights (shoot, root, flower and total) of plant in both HMBC and MBC-SAP treatments were higher than those in MBC treatment (*p* < 0.05). Till 35 d, the total dry weight of cabbage was 930.10 ± 22.89, 704.43 ± 16.17 mg/plant in HMBC and MBC-SAP treatments, separately. Meanwhile, the dry weight of the edible leafy portion (shoot part) of cabbage was significantly higher in HMBC treatment than those in MBC-SAP and MBC control (*p* < 0.05), with 571.97 ± 21.40 mg/plant contrast to 398.27 ± 17.59 mg/plant and 365.23 ± 11.31 mg/plant, respectively. We also found the similar result in S_3_. The biomass of cabbage in HMBC treatment was significantly higher than those in the MBC-SAP and MBC treatments, with the highest height for 25.14 ± 0.35 cm, dry weight of shoot for 443.43 ± 23.50 mg/plant and the total dry weight for 675.40 ± 29.07 mg/plant at day 35. However, for the MBC-SAP treatment in S_3_, the biomass of cabbage was lower than the MBC control but statistically equal to those of the blank control without MBC-BR, with the lowest height for 18.32 ± 0.59 cm, dry weight of root for 110.73 ± 7.07 mg/plant and the total dry weight for 427.86 ± 18.79 mg/plant at day 35.

### The distribution of ^14^C radioactivity in cabbage in soil amended with BR of MBC

**Figure 1:**
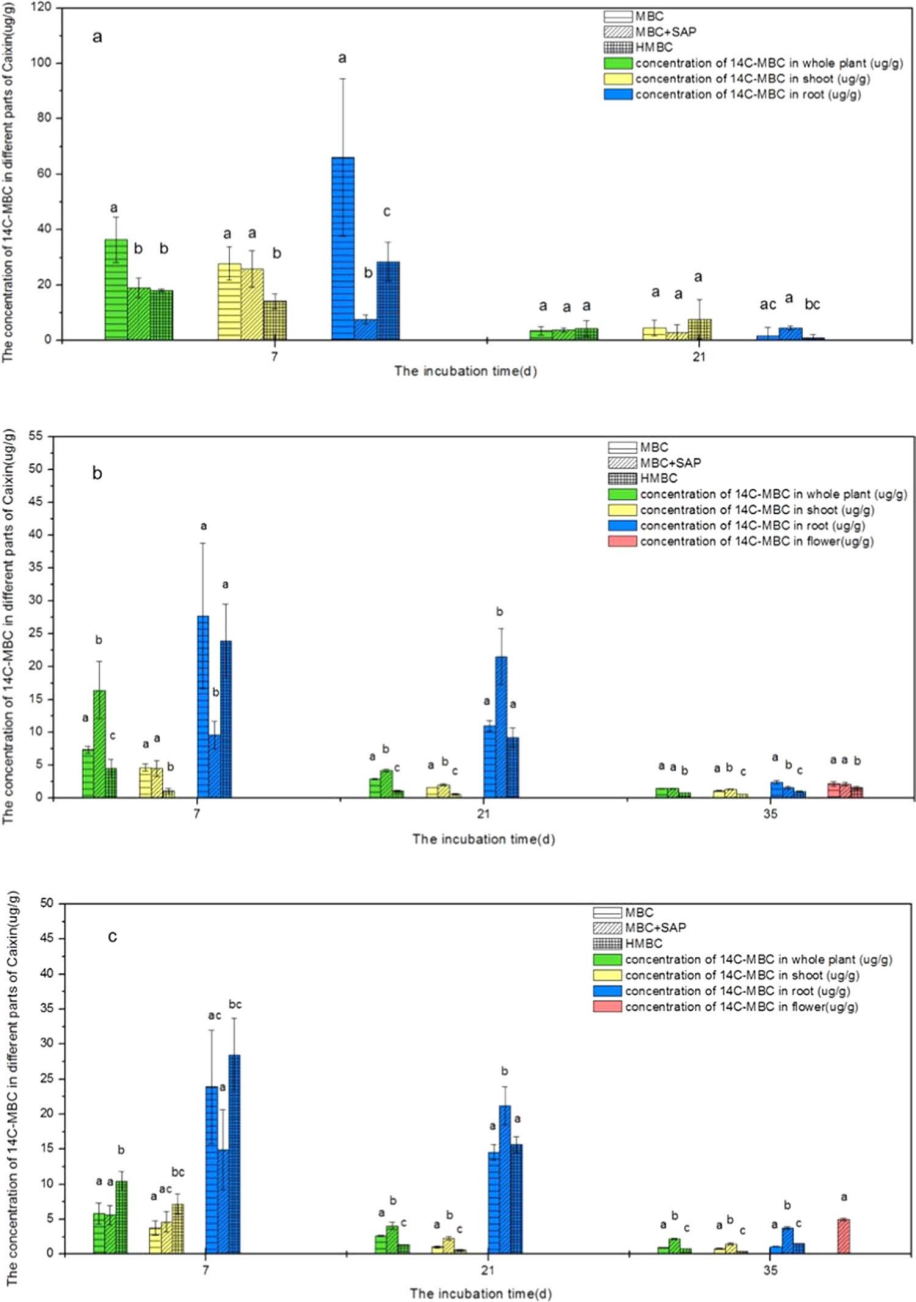
The distribution of ^14^C radioactivity in Caixin seedling grown in soil amendment with BR of Carbendazim. (**a**) soil 1 (S_1_); (**b**) soil 2 (S_2_); (**c**) soil 3 (S_3_).

During the cabbage growth in BR spiked soil, the ^14^C radioactivity in vegetable tissues were detected, which means cabbage could absorb the ^14^C-compound from soil spiked with released BR. Analysis of ^14^C radioactivity in cabbage showed the bioaccumulation of ^14^C content in shoot, root and the whole plant were mostly higher in the initial incubation than those in the final phase planting (Fig. 1). Figure 1a shows the ^14^C distribution in cabbage in S_1_. Obviously, a majority of ^14^C content was accumulated in root, and less ^14^C existed in shoot, suggesting the root was the main enrichment site of soil released BR. Compared with MBC control (36.50 ± 8.14 μg/g), the ^14^C content of whole plant was significantly lower in MBC-SAP and HMBC treatments at 7 d, with the corresponding values 19.02 ± 3.46 μg/g and 18.07 ± 0.55 μg/g on the dry biomass basis, separately. For the shoot, ^14^C level in HMBC treatment was significantly lower than those in the MBC-SAP and MBC treatment (*p* < 0.05). While, for the ^14^C accumulation in root, there was no difference between the HMBC treatment and MBC control (*p* > 0.05), those much higher than that in MBC-SAP treatment. However, when the cabbage planted until 21 d, no significant difference was observed between three different treatments for the ^14^C distribution in plant.

However, cabbage could grow vigorously and blossomed in 35 d in S_2_. We detected the ^14^C accumulation in the flowers part (Fig. 1b). The ^14^C content of flowers was 2.14 ± 0.33 μg/g, 2.10 ± 0.23 μg/g and 1.53 ± 0.23 μg/g in MBC, MBC-SAP and HMBC treatments, respectively. There was a decrease of ^14^C-bioaccumulation in whole plant, shoot and root of cabbage with the extension of incubation. When cabbage was cultured for 35 days, the ^14^C-distribution in whole plant was decreased from the initials 4.61 ± 0.53 ug/g to 1.04 ± 0.08 ug/g for MBC treatment, 4.49 ± 1.23 μg/g to 1.30 ± 0.07 μg/g for MBC-SAP treatment, 1.05 ± 0.46 to 0.58 ± 0.03 μg/g for HMBC treatment, separately. Obviously, the ^14^C accumulation in whole plant was the lowest in HMBC treatment. However, in MBC-SAP treatment, the cabbage could absorb much more released BR from soil in whole tissue during the incubation, with 16.41 ± 4.33 μg/g, and 4.19 ± 0.19 μg/g at 7d and 21d, separately, compared with MBC control (7.35 ± 0.58 μg/g and 2.90 ± 0.12 μg/g). For the root accumulation, there was no significant difference between the HMBC and MBC treatments during the 21 days incubation (*p* > 0.05). While the ^14^C content in MBC-SAP treatments decreased firstly then increased at 21 d. At 100d, the ^14^C accumulation in root was followed in order of MBC > MBC-SAP > HMBC treatment.

Similarly, we found the same trend of ^14^C bioaccumulation in cabbage in S_3_. An increase in ^14^C content in whole plant was consistent with the decrease in plant height and dry weight. At 7 d, the ^14^C content of whole plant in HMBC treatment was higher than in the MBC-SAP and MBC treatments corresponding to the lower biomass of total plant (Table 1 & Fig. 1c). This suggested the plant growth inhibition was due to the accumulation of the released chemicals from BR of MBC in soils. When cabbage cultured at 21 d and 35 d, the whole plant ^14^C in MBC-SAP was higher than those in MBC and HMBC treatments. For instance, when cultured at 35d, the ^14^C level in whole plant was 2.19 ± 0.11 μg/g in MBC-SAP treatment, significantly higher than the MBC (0.92 ± 0.07 μg/g) and HMBC treatment (0.75 ± 0.02 μg/g), respectively (*p* < 0.05). HMBC treatment has the lowest ^14^C accumulation in the whole plant among all the treatments. We also have the similar change trend for ^14^C bioaccumulation in shoot, with an order in HMBC < MBC < MBC-SAP treatment. For the root, the ^14^C activity reached a maximum at 21 d in the MBC-SAP treatment (21.21 ± 2.70 μg/g), which was significantly higher than the MBC (14.59 ± 1.14 μg/g) and HMBC treatments (15.70 ± 1.15 μg/g) (*p* < 0.05).

### Determination of released BR in different SAP amendment treatments

**Figure 2:**
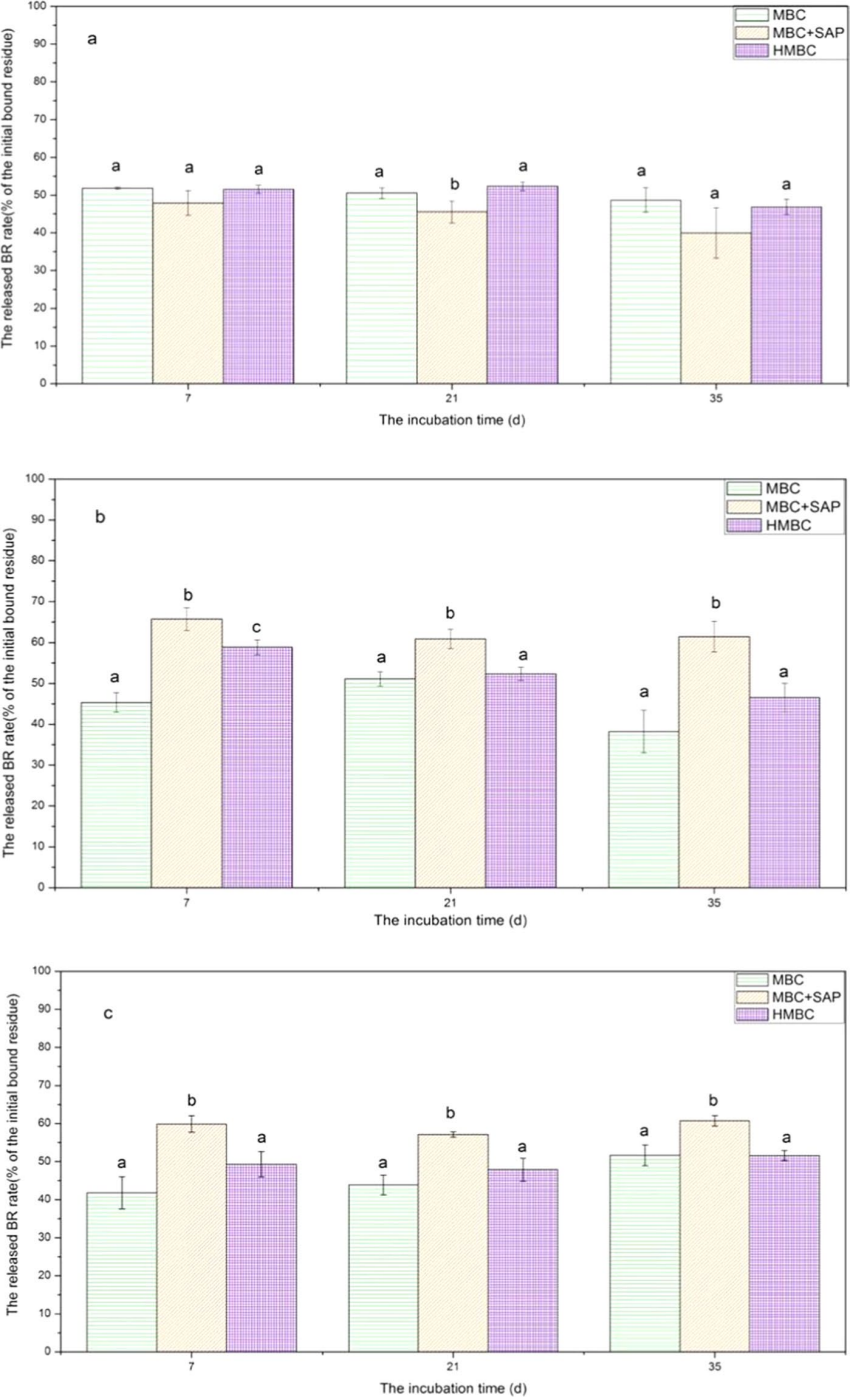
The released bound residue (BR) in soil after sowing Caixin. (**a**) soil 1 (S_1_); (**b**) soil 2 (S_2_); (**c**) soil 3 (S_3_).

After the cabbage cultivation and the subsequent extraction of soil, the decreased radioactivity in whole soil was the fraction of the released BR. The fraction of BR release after cabbage planting was 51.87 ± 0.28%, 47.91 ± 3.34%, and 51.57 ± 1.07% of the initial applied activity in S_1_, respectively for MBC, MBC-SAP and HMBC treatment at 7 d (Fig. 2a). There was no significant change of the released BR during the incubation in each BR-amended treatment (*p* > 0.05).

**Figure 3:**
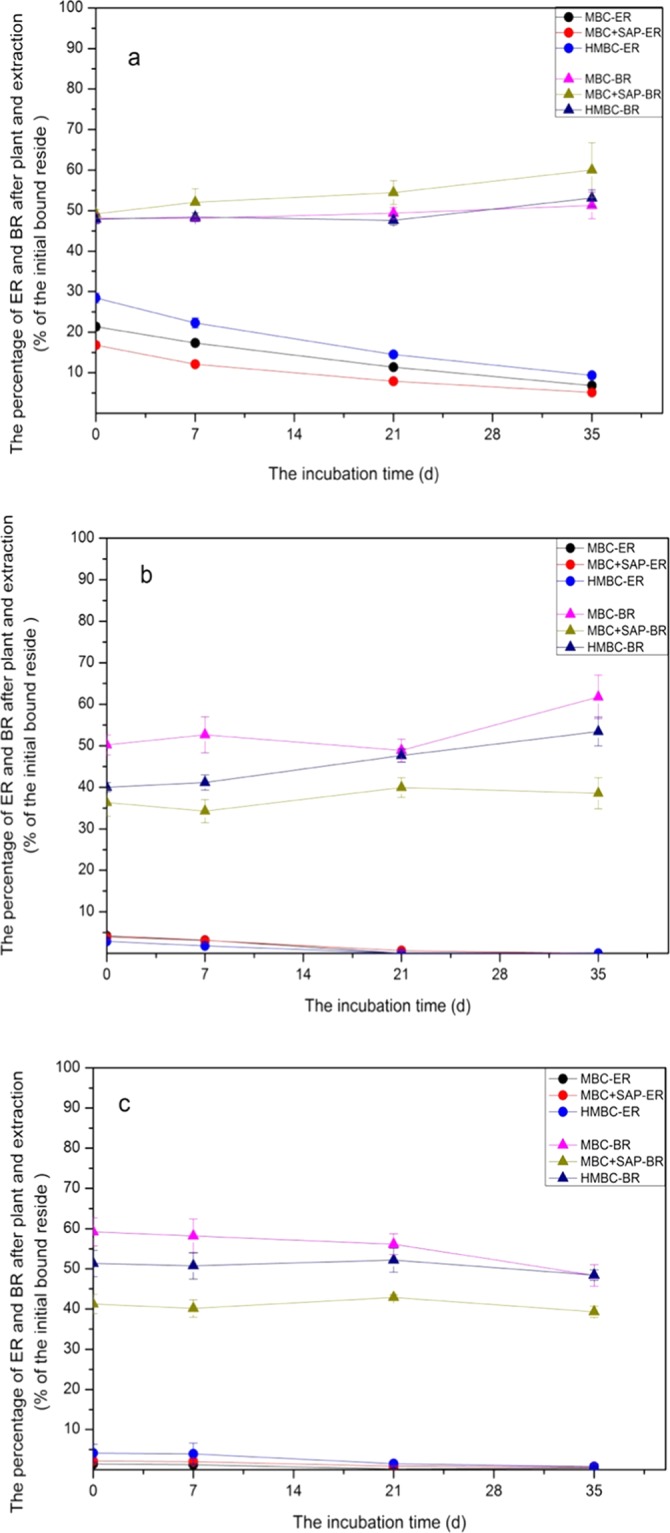
The extractable residue and bound residue in soil after sowing Caixin and extraction. (**a**) soil 1 (S_1_); (**b**) soil 2 (S_2_); (**c**) soil 3 (S_3_).

In additional, the soils amended with MBC-BR after cabbage culture was extracted, and the extractable part was combined and nominated as extractable residue (ER). ER decreased with incubation time, and there was significant difference between these three treatments (*p* < 0.05), with following the order of HMBC > MBC > MBC-SAP (Fig. 3a) in S_1_. Compared to MBC control, more ER was detected in HMBC treatment, corresponding to the lower ^14^C bioaccumulation in plant mostly concentrated on the root. The lowest amount of ER was found in MBC-SAP treatment. Furthermore, there were still high amount of BR in soil after extraction and cabbage culture at the end of incubation, with 51.32 ± 3.29%, 60.03 ± 6.69% and 53.12 ± 2.04% respectively, for MBC, MBC-SAP and HMBC treatment (Fig. 3a). There was no significant difference between three treatments (*p* > 0.05).

In S_2_, a little ER was extracted from soil after cabbage planting during the incubation, with approximately 0.13~3.17% of the initial BR amount (Fig. 3b). This suggested mostly released BR was taken up by plant or turned into BR or released as CO_2_ into atmosphere. During incubation, the lowest amount of BR was found after extraction in the MBC-SAP treatment (38.57 ± 3.75%) when compared with MBC and HMBC treatment (61.76 ± 5.26% and 53.44 ± 3.46%, respectively). But much larger amount of the released BR was calculated in MBC-SAP treatment, with the relative enhancement of 37.74% and 24.21%, compared to the MBC treatment and HMBC treatment, respectively at 100 d (Fig. 2b). Similarly, we detected the same result in S_3_ in terms of released BR rate. The released rate of BR was 51.66 ± 2.70%, 60.70 ± 1.40%, and 51.57 ± 1.29%, respectively in MBC, MBC-SAP and HMBC treatment at 35 d (Fig. 2c). Larger amount of released BR in MBC-SAP treatment may result in the higher amount of ^14^C per dry biomass of cabbage, and the inhibition of cabbage growth, corresponding to our above results. However, there was no significant difference between MBC and HMBC treatment in terms of released BR rate (*p* > 0.05). In additional, at the end of incubation, the amount of soil BR after extraction and cabbage growing was 48.34 ± 2.70%, 39.30 ± 1.39% and 48.43 ± 1.29% in MBC, MBC-SAP and HMBC treatment, respectively. Obviously, the lowest amount of BR existed in MBC-SAP treatment.

## Discussion

Superabsorbent hydrogel (SAP) usually are used as soil conditioners and chemical formulations in agriculture to maintain soil moisture and enhance the pesticide efficiency^17^. However, some research showed when pesticides coexisted with SAP, the environmental fate and transformation of pesticides could be changed, especially for bound residue^12,13,18^. The bound residue could be released during further agricultural practices and may influence the succeeding crop and soil management^2,19^.

In this study, we studied the plant availability and phytotoxicity of MBC-BR in three typical cultivated soils amended with SAP and SHs-formulations. Different SAP amendments could affect the cabbage growth differently, including plant height, dry weight of shoot, root, flower, and total plant. For acidic clayey soil S_1_, cabbage grew till 21 d, with lowest plant height and dry weight of whole plant compared with others soils, with no significant difference between MBC, MBC-SAP and HMBC treatment during the incubation (*p* > 0.05). This is might be due to S_1_ with barren microbes and low pH value and electrical conductivity, which is not favorable for the planting crops, and cabbage could not grow healthily. While for neutral loamy soil S_2_ and basic saline soil S_3_ with rich microbes, organic matter and high electrical conductivity, cabbage underwent the favorable growth, and there was the highest plant height and dry weight of whole plant in HMBC treatment when compared with MBC treatment. MBC-BR in SHs-encapsulated treatment and SAP amendment treatment could enhance the growth of cabbage in S_2_, especially for SHs-encapsulated treatment. SAP, as soil conditioner, could retain water and keep soil moisture, and hold the water for the plant growing during incubation. In addition, SAP could be a favorable nutrition sources for soil microorganism. It might enhance the activity and biodiversity of microbes to some extent^20,21^. But for MBC-SAP treatment, the growth of cabbage was inhibited during the incubation, especially for S_3_. However, different forms of SAP amendments could affect cabbage growth in BR amended soil differently. The different bio-effect on cabbage in soil might also related with the different BR release rates. Han *et al*. demonstrated the plant height and dry weight decreased as BR amendment increased, and the herbicide ZJ0273 and its metabolites from released BR imposed serious phytotoxic effects on rice plant^3^.

The dry weights of cabbage shoot and whole plant in HMBC treatment were increased substantially compared with MBC-SAP and MBC alone treatment in all soils. Based on previous results, SHs-encapsulated formulation could significantly increase the dissipation and mineralization of MBC, and reduce the BR substantially^13^. With the environment friendly transformation fate of HMBC and favorable biomass for cabbage, SHs-encapsulated formulation might be a good way to spike the pesticide into the environment and cut down the potential hazards to agro-ecosysterm.

Meanwhile, the ^14^C-distribution in cabbage was detected in three treatments, there was an increase of the ^14^C-bioaccumulation in cabbage whole plant, shoot and root with the incubation in all treatments. The ^14^C bioaccumulation of cabbage is closely related with the biomass of cabbage and the amount of released BR during the incubation. ^14^C content in whole plant and shoot part of cabbage were the lowest in HMBC treatment compared with MBC and MBC-SAP treatments, suggesting HMBC could reduce the released BR accumulation in cabbage, especially for the edible shoot. However, cabbage could absorb much more released BR from soil in whole tissue and edible part of cabbage during the incubation in MBC-SAP treatment. According to the European Food Safety Authority, the maximum residue level (MRLs) of MBC in sugar beets and vegetable is <5 mg/kg in vegetable. For cabbage cultured at initial stage in neutral soil S_2_ and basic soil S_3_, the ^14^C-bioaccumulation in plant and edible part has been exceeded the standard value, and are higher in MBC-SAP than MBC and HMBC treatment. The acceptable daily intake (ADI) index is 0.02 mg/kg bw/d (body weight per day). So for an adult with 100 kg, the maximum ingestion of MBC per day is 2 mg. Thus it is important for us, and we should give priority attention to the food safety especially for crop grown in the soil spiked with SAP. It seems HMBC may be the potential way to spike pesticides into environment without the high pesticide residue bioaccumulation in cabbage and with safe fate in ecological environment^12,13^.

Based on BR release results, we detected higher released BR in MBC-SAP treatment. This plant growth inhibition might be due to the accumulation of the released chemicals from MBC-BR in soils. Different SAP amended treatments could trigger the remarkable different effects on soil BR release after sowing the succeeding crops. Based on our previous results of the fate of carbendazim amended with SAP and SHs-formulation, there was still higher amount of initial carbendazim BR in MBC-SAP treatment compared with MBC control and HMBC^12,13^. It seems that when we spike the carbendazim and SAP amendment into soil, there will be much more BR in soil and this residue is more readily released when we planted cabbage in the BR-amended soil. Compared with HMBC treatment, the MBC-SAP treatment did not have safer environmental effect in soil and crops. Gevao *et al*. indicated BR can be released by physicochemicals mechanism or through biochemical process^22^. Agriculture practices and the introduction of certain chemicals that may change soil texture and property could result in BR releasing. SAP could change the soil texture and keep moisture, and also alter the soil microorganism abundance and biodiversity^23^. Physical entrapment of SAP, MBC, and metabolites in soil organic and inorganic matrices stimulated by microorganisms could lead to the formation of organoclay complexes and soil aggregates, with the soil BR increasing during the incubation. Meanwhile, SAP also could interact with the surface active of MBC and its metabolites due to some polar groups of –OH, –COOH, and –NH_2_^22^. These physical and chemical changes could alter the BR substance and may be released upon planting crops. Gao *et al*. suggested the plant root exudates usually play an important role in the environmental processing of organic pollutants, which could release the BR in soils^4^.

After the cabbage incubation, the soil bound residue differed in different SAP amendments and soils, following the order of MBC-SAP < HMBC = MBC treatment at the end of incubation in the neutral soil S_2_ and basic soil S_3_. Though BR was low in soil in MBC-SAP treatment, we should pay more attention on the further BR release in terms of succeeding crops and soil management. Above all, compared to the MBC treatment, cabbage could accumulate the released BR from soil more easily in MBC-SAP treatment, and less in HMBC treatment. This behavior might be closely related with the amount of BR which was released from the soil during the plant culture.

## Conclusion

Our findings show that high attention should be given to the environmental risk assessment of pesticides BR when pesticides spiked with SAP amendments. When SAP is amended into the soil environment to keep soil moisture and enhance pesticides efficiency, in comparison, the SHs-formulation seems safer than SAP amendment for the utility of pesticides in the environment. SHs-encapsulated formulations may be a promising efficient and environmentally friendly method to reduce pesticide residue and keep the natural ecosystem and human health. In addition, the toxicity effects and long-term stability of other pesticides-SHs encapsulated formulations also need to be carried out to achieve the safely exploitation in further research.

## Materials and Methods

### Chemicals and reagents

**Figure 4:**
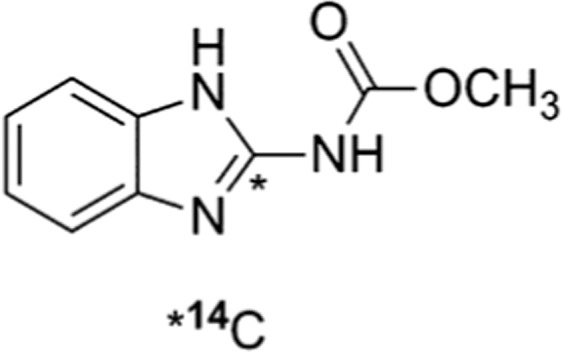
The structure of ^14^C-Carbendazim.

^14^C-MBC (methyl-2-benzimidazole carbamate), with ^14^C-labeled imidazole ring (Fig. 4) was obtained from ChemDepo Incorp. (Camarillo, CA). The radiochemical and chemical purity of ^14^C-MBC was >97% and specific activity was 1.89 × 10^9^ Bq mmol^−1^. Non-labeled MBC (chemical purity >96%) was purchased from Sigma-Aldrich (Munich, Germany). The ^14^C-MBC stock solution was prepared by mixing the labeled Carbendazim and non-labeled in methanol at a final specific activity of 4.625 × 10^4^ Bq mg^−1^. Acetonitrile and glacial acetic acid were HPLC grade agents. All solvents such as hydrochloric acid, ethanolamine, sodium hydroxide, methanol, and glycol ether were of analytical grade. The cocktail A solution contained 0.5 g of 1,4-bis (5-phenyloxazoly-2-yl)-benzene (POPOP), 7.0 g of 2,5-diphenyloxazole (PPO), 650 mL of dimethyl benzene and 350 mL of glycol ether. Cocktail B contained 0.5 g of POPOP, 7.0 g of PPO, 550 mL of dimethyl benzene, 275 mL of glycol ether, and 175 mL of ethanolamine.

### Soil, SAP and H-^14^C-MBC

Soils were sampled from the first horizon (0–15 cm) in agricultural fields in Hangzhou (fluvio-marine yellow loamy soil), Cixi (coastal saline soil), and Longyou (red clay soil), Zhejiang Province, China, which are abbreviated separately herein as S_1_, S_2_, and S_3_. All soils were air-dried, sieved through a 2 mm mesh and stored at room temperature before use. The main physico-chemical properties of soils were determined using standard methods and summarized in Table 2.

**Table 2:** Main physicochemical properties of soils.

Property	Soil type		
	S_1_	S_2_	S_3_
	Red sandy clay soil	Fluvio-marine yellow loamy soil	Coastal saline soil
Location	Longyou, Zhejiang	Hangzhou, Zhejiang	Cixi, Zhejiang
pH(H_2_O)	4.20	7.02	8.84
Clay (%)	39.0	8.0	24.3
Silt (%)	41.1	71.2	71.1
Sand (%)	19.9	20.8	4.6
P(mg kg^−1^)	3.21	25.20	10.80
K(mg kg^−1^)	4650	8122	9768
Ca(mg kg^−1^)	4321	7790	9420
Mg(mg kg^−1^)	3238	6949	8284
S(mg kg^−1^)	4.91	30.21	12.87
OM^a^ (g kg^−1^)	8.40	30.50	9.50
CEC^b^ (cmol kg^−1^)	6.62	10.83	10.17
Total N (%)	0.34	2.90	1.80
WHC^c^ (%)	33.2	33.7	37.1

^a^Organic matter; ^b^Cation exchange capacity; ^c^Water-holding capacity.

SAP, starch-graft-polyacrylamide (St-g-PAM) superabsorbent cross-linked by N,N-methyl bisacrylamide were synthsized via 10 MeV simultaneous electron beam irradiation at room temperature and subsequent alkaline hydrolysis. The swelling ratio of SAP (deionized water) was approximately 1000 g g^−1^. SAP was dried at room temperature in a vacuum drying apparatus^24^.

The H-^14^C-MBC was prepared using dry starch-g-polyacrylamide and starch-*g*-(acrylic acid-*co*-methyl methacrylate) N,N’-methyl bis-acrylamide, following the polymerization reaction at 85 °C for 30 min under N_2_ atmosphere to form the gelatinized starch. A predetermined quantity of carbendazim dissolved in acrylic acid was mixed with a small part of starch paste in a temperature-controlled water bath and stirred (300 rpm), and heated to 70 °C, was added by 0.05 g of AIBA(2’2-azobis[2-methylpropionamidine]dihydrochloride]). This reaction was proceeding for 5 h under a N_2_ atmosphere with reflux condensation to form the mixture A. Another part of starch was taken to synthesize the hydrogels (mixture B). The two mixtures were blended, and heated up to 70 °C until to obtain the rubbery product. Finally, the H-^14^C-MBC was dried, and sieved for incubation experiment^25^.

### Incubation experiment and preparation of the bound residue

After 10 days pre-incubation, three 300 g aliquots of each soil was amended with SAP (0.5‰ (w/w)), and then were mixed with MBC at 4 mg kg^−1^. Subsequently, soil moisture content was regulated to 60% of water-holding capacity (WHC). Similarly, the SAP-free treatment with Carbendazim and SAP-encapsulated formulations underwent the same procedure. The incubation test was performed under aerobic conditions according to OECD (2002) guideline 307^26^. The fully mixed soils were transferred to 500-mL brown jars connected by a flow-through apparatus to the trapping solutions. All treatments were incubated at 25 ± 1 °C and ventilated periodically, and all absorption solutions were exchanged regularly with fresh solutions. At intervals of 0, 3, 6, 13, 20, 30, 45, 60, 80, and 100 d, three replicates of each treatment (10 g, dry weight equivalent) were collected.

Soil samples (10.0 g, dry weight) per treatment were extracted sequentially according to Helweg and Wang *et al*. with slight modification^27,28^. Briefly, soil samples were extracted three times with 30 mL of methanol/0.1 M hydrochloric acid solution (4:1, v/v), blended thoroughly, and shaken at 120 rpm for 2 h. After centrifugation at 6000 × *g* for 5 min, the deposits were similarly re-extracted by methanol, and ethyl acetate, consecutively, until no more ^14^C-radioactivity was detected in the extracts. The recovery extraction of ^14^C activity was approximately 95.52–101.65% when freshly spiked soils were analyzed. A 1-mL aliquot of every treatment supernatant was measured with addition of 10-mL cocktail A to measure the ^14^C-activity on LSC. The ^14^C-radioactivity of total extracted solvents was calculated as the extractable residue (ER). All remaining solutions were passed through a 0.22-μm filter and reduced in bulk to near dryness by a Vacuum Rotary Evaporator (Eyela SB-1000, Eyela, Tokyo, Japan) at 45 °C. The residue was re-dissolved in 10-mL methanol and condensed to 1-mL under a stream of nitrogen at ambient temperature for high performance liquid chromatography-tandem mass spectrometry (HPLC-MS/MS) analysis. All the post-extracted soils were air-dried. A homogenized soil sample of 1.0 g was combusted on the biological oxidizer and the released ^14^C-CO_2_ was trapped in 15 mL of cocktail B for analysis on LSC. The combustion recovery was 95.70 ± 1.42% (n = 3). The amount of ^14^C-radioactivity in the post-extracted soils was defined as the initial bound residue (BR).

### Bioavailability experiment

Flowering Chinese cabbage was used for the bioavailability assay. The initial BR soil was mixed with fresh soils at the initial contents of ^14^C-BR of carbendazim (Table 3).

**Table 3:** The initial contents of ^14^C-BR of carbendazim in sowing soil.

Soil	Treatment	BR content (%) of the ^14^C applied amount
S_1_	MBC	33.98 ± 1.81
	MBC-SAP	40.50 ± 1.12
	HMBC	45.90 ± 2.22
S_2_	MBC	69.76 ± 2.07
	MBC-SAP	77.66 ± 2.66
	HMBC	59.23 ± 3.28
S_3_	MBC	74.12 ± 2.10
	MBC-SAP	82.33 ± 1.43
	HMBC	63.61 ± 2.20

The uniformly mixed soil (50 g, dry weight equivalent) were placed in each 100-mL plastic pot for cultivation. The moisture of soil was adjusted to 60% of the soil WHC. Each germinated seeds were sown in each pot. Blank soils without ^14^C-BR soil were also planted with seeds as above. All treatments were incubated also under the same green conditions (25/20 °C, day/night; humidity, 80%; light, 16 h/8 h), with daily irrigation. The cabbage seedling for each treatment was harvested at 7, 21 and 35 days of exposure. The shoots and roots of seedling were separated. The roots were washed with tap water, and the height of plant was measured. All the plant parts were kept in paper envelope and dried at 60 °C to a constant weight. Aliquots of five dried plants were combusted on the biological oxidizer, and the released ^14^CO_2_ was absorbed in 15 mL liquid scintillation cocktail B. The radioactivity was measured by Quantulus 1220 ultra-low liquid scintillation spectrometer (ULLSS; Quantulus 1220, Perkinelmer, Turku, Finland) to estimate the amount of BR that was accumulated by the plant. The recovery efficiency of the above combustion procedure was 93.32 ± 1.41%.

### Measurement of the released bound residue

After the cultivation of Cabbage, the soils were extracted by the same method. Aliquots of the final extract at each extraction step were transferred into 20-mL scintillation vials, and the ^14^C radioactivity was measured by LSC after addition of 10-mL scintillation cocktail A. Then all extracts were mixed together and condensed to near dryness on a vacuumed rotary evaporator (Eyela SB-1000, Eyela Co. Shanghai, China) at 40 °C. The residue was dissolved in 1.0 mL methanol, and the ^14^C-radioactivity of total extracted solvents was calculated as the extractable residue (ER). All the post-extracted soils were air-dried. 1.0 g homogenized soil sample was combusted on biological oxidizer and the released ^14^C-CO_2_ was trapped in 15 mL of cocktail B for analysis on LSC. The combustion recovery was 95.70 ± 1.42% (n = 3). The amount of ^14^C-radioactivity in the post-extracted soils was defined as bound residue (BR). The released rate of bound residue was calculated as initial BR minus the BR after the plant seeding, then divided by the initial BR.

1 $${\rm{Release}}\,{\rm{rate}}=({{\rm{BR}}}_{{\rm{i}}}-{{\rm{BR}}}_{{\rm{a}}})\ast 100 \% /{{\rm{BR}}}_{{\rm{i}}}$$

### Statistical analysis

All statistical analysis was performed using Origin 8.0 (Microcal Software, Northampton, MA) and SPSS 20.0 (IBM SPSS Statistics, Armonk, NY, U. S. A.). The significance was based on one-way ANOVA at α = 0.05. The data were presented as the mean ± standard derivation of three replicates.
